# Supplementary material for: How should trauma discussions be approached in maternity care? Perspectives from a qualitative study with women, voluntary sector representatives and healthcare providers in the UK
Source: BMJ Open. 2025 Dec 3;15(12):e097815. doi: 10.1136/bmjopen-2024-097815 (PMC12706108; doi:10.1136/bmjopen-2024-097815)
Supplement: Supplementary data [file bmjopen-15-12-s001.pdf]

Supplementary material

Topic guide for all interviewees

HOW SHOULD MATERNITY CARE PROVIDERS ASK ABOUT DIFFICULT PAST EXPERIENCES?

1. Do you think maternity care providers **should** ask pregnant women about difficult past experiences?

2. **When** should maternity care providers ask about difficult experiences?

PROMPTS:

- At booking?
- At a later routine appointment?
- At a separate appointment for this purpose?
- At multiple appointments?
- Is continuity of care / existing relationship important?

3. **Who** should ask about difficult experiences?

PROMPTS:

- Is professional background important (midwife / care assistant / obstetrician?)
- Is the gender of the person asking important?

4. **Where** should these discussions take place?

PROMPTS:

- Is this important?
- Home / clinic - hospital or community setting?
- What if partners / children are present?

Cull J, *et al. BMJ Open* 2025; 15:e097815. doi: 10.1136/bmjopen-2024-097815

5. How can maternity care providers ensure that women who want to discuss their histories **feel comfortable** to do so?

PROMPTS:

- How can the questions be asked sensitively?
- Should partners be excluded from part or all of an appointment to allow these issues to be discussed in private?
- For women who want to disclose their histories, what do you think would prevent them from doing so?

6. **How** should maternity care providers ask about difficult past experiences?

PROMPTS:

- How should the question be asked - direct question? General discussion? Questionnaire?
- Show examples:
  - Antenatal Psychosocial Risk Questionnaire (ANRQ) (Austin et al., 2013)
  - ACE-10 Questionnaire (Felitti et al., 1998)
  - Kimberley Mum's Mood Scale (Marley et al., 2017)
  - Trauma History Questionnaire (Green, 1996)
  - Hypothetical Prompt developed by White, Danis and Gillece (2016)
  - Prompt developed by Montgomery (does not explicitly name abuse) 'Sometimes pregnancy can trigger unexpected memories of things that have happened to you or feelings that can take you by surprise. If that happens to you and you would like to talk about it, please let me know'.
- Complete through conversation with maternity care provider, or self-complete (on ipad or paper) then discuss with maternity care provider?

- Another option is to complete independently from maternity care, e.g. online tool which encourages women to seek support from healthcare provider but also provides links to relevant third sector agencies
- How can the questions be asked sensitively?

7. How should maternity care providers **prepare women** for this conversation and let them know the purpose of the discussion?

PROMPTS:

- What are the issues around confidentiality?
- Should we talk with women about potential negative implications of disclosures (for example, if children / self potentially at harm maternity care provider will need to share this information)?
- Should we talk with women about the potential positive implications of disclosure? To receive support and understanding, be offered adaptations to care, signpost or refer to services that might help in healing.

8. Are there any additional considerations when discussing trauma with **women who don't speak English as a first language**?
    - Challenges around use of interpreters
    - Looking back at methods of discussing trauma with women (e.g. Kimberley Mums Mood Scale), how easy would they be to understand for women with limited English?
    - Would women prefer to self-complete trauma checklist in own language?

9. Some of the **language used around difficult past experiences** may make these conversations harder for women. Are there any terms that you think maternity care providers should avoid?
PROMPTS
    - Examples - victim / survivor
    - Trauma / abuse / difficult experiences

- Maternity care providers have said that when they're talking about caring for women who have experienced trauma, they're not sure how to refer to them. For example, survivor moms. Do you have any thoughts about this?

HOW SHOULD MATERNITY CARE PROVIDERS RESPOND TO DISCLOSURES OF PREVIOUS TRAUMA?

10. How should the information be **recorded and shared**?

PROMPTS:

- Within the maternity team
- With wider support services e.g. Health Visitor, GP, neonatal team, perinatal mental health, safeguarding, third sector
- Consent for this
- Limits of confidentiality
- Electronic / hand-held record

11. What **information and support** might women who have had difficult experiences find helpful?

PROMPTS:

- For example mental health specialists / support from the voluntary sector
- Are there any other helpful resources you are aware of, for example books or websites?
- Do you think tailored small group antenatal classes for women who have had difficult experiences would be beneficial? (like Centering Pregnancy)
- How about peer or lay support groups?
- Or groups with more of a social focus, not explicitly about trauma but offering arts / movement / meditation?
- Using a friends and family diagram (genogram) - strengths based approaches to help women identify the sources of support in their lives and communities?

- Can you think of any services which aren't currently offered, but would be helpful to women?

12. How can **maternity care be adapted** to help women who have had difficult experiences?

PROMPTS:

- E.g. continuity of carer, limited vaginal examinations, elective caesarean section

13. What do you think should be included in **training for maternity care providers** around trauma-informed care?

PROMPTS:

- Training in communication skills to sensitively ask about previous trauma and respond to disclosures?
- Instances in which safeguarding procedures will, and will not, have to be followed, and how to discuss these with women.

14. **What difference** do you think discussing prior trauma with women and providing support could make?
    - What matters to women?
    - What should we be measuring?
15. Is there anything I haven't asked that you would **like to add**?

PROMPT

- Do you think specific conditions need to be in place before routine discussion of trauma is introduced?
- Was it helpful to receive the topic guide in advance?

ADDITIONAL QUESTIONS FOR HEALTHCARE PROFESSIONALS AND VOLUNTARY SECTOR EXPERTS ONLY

16. How can **adequate time be ensured** for these conversations to be meaningfully had within an overstretched and understaffed service?
17. How can women who have experienced trauma be recognised through **verbal and non-verbal signals**?

PROMPTS:

- How can care providers support women who they suspect are experiencing the effects of trauma, but have chosen not to disclose?

- Some providers have talked about 'universal precautions': assuming all women have experienced trauma as it is so prevalent. How do you think care providers can adjust their care to avoid causing distress to women who have had difficult past experiences?

18. Trauma disclosures can be distressing to hear. How can **midwives' emotional wellbeing be protected**?

PROMPTS:

- Supervision models - Who should provide the support - a midwife within the trust (Professional Midwifery Advocate) or a trained therapist? Should this be on an as-requested basis or with regular meetings (automatic supervision)?
- Midwives who have been, or are currently in, violent or abusive situations may find training around this issue distressing, and may find these conversations difficult. How can they best be supported?
- Should midwives be able to opt out of having these discussions? Some midwives may not have the personal resources to support women around these issues due to their own trauma.
